# Genetic Diversity Does Not Contribute to Attenuation of HeLa Passaged Wild-Type Yellow Fever Virus Strain French Viscerotropic Virus

**DOI:** 10.3390/v14030527

**Published:** 2022-03-04

**Authors:** Ashley E. Strother, Jill K. Thompson, Steven G. Widen, Alan D. T. Barrett

**Affiliations:** 1 Department of Pathology, University of Texas Medical Branch (UTMB), Galveston, TX 77555, USA; aestroth@utmb.edu; 2 Department of Biochemistry and Molecular Biology, University of Texas Medical Branch (UTMB), Galveston, TX 77555, USA; jkthomps@utmb.edu (J.K.T.); sgwiden@utmb.edu (S.G.W.); 3 Sealy Institute for Vaccine Sciences, University of Texas Medical Branch (UTMB), Galveston, TX 77555, USA

**Keywords:** attenuation, flavivirus, yellow fever virus, genome, genetic diversity

## Abstract

The disease yellow fever was prevented by two live attenuated vaccines, strains 17D and French neurotropic vaccine (FNV), derived by serial passage of wild-type (WT) strains Asibi and French Viscerotropic virus (FVV), respectively. Both 17D and FNV displayed decreased genetic diversity and resistance to the antiviral Ribavirin compared to their WT parental strains, which are thought to contribute to their attenuated phenotypes. Subsequent studies found that only a few passages of WT strain FVV in HeLa cells resulted in an attenuated virus. In the current study, the genome sequence of FVV following five passages in HeLa cells (FVV HeLa p5) was determined through Next Generation Sequencing (NGS) with the aim to investigate the molecular basis of viral attenuation. It was found that WT FVV and FVV HeLa p5 virus differed by five amino acid substitutions: E-D155A, E-K331R, E-I412V, NS2A-T105A, and NS4B-V98I. Surprisingly, the genetic diversity and Ribavirin resistance of the FVV HeLa p5 virus were not statistically different to WT parent FVV. These findings suggest that while FVV HeLa p5 is attenuated, this is not dependent on a high-fidelity replication complex, characterized by reduced genetic diversity or increased Ribavirin stability, as seen with FNV and 17D vaccines.

## 1. Introduction

Yellow fever virus (YFV) is the prototypical member of the genus *Flavivirus* [1]. The virus has a single-stranded, positive-sense RNA genome approximately 11 kb in length that encodes three structural (capsid, membrane [M], and envelope [E]) and seven non-structural proteins (NS1, NS2A, NS2B, NS3, NS4A, NS4B, and NS5). Mosquito-borne YFV is endemic in tropical South America and sub-Saharan Africa. The viscerotropic disease, yellow fever (YF), caused by wild-type YFV is characterized by infection of the liver, resulting in the onset of jaundice in severe cases for which the disease and virus are named [2,3,4,5]. Two live attenuated vaccines (LAVs) were developed concurrently in the 1930s to prevent YF. The vaccine currently used today, strain 17D, was derived by 176 serial passages of wild-type (WT) strain Asibi in mouse tissue and chicken embryo tissue both with and without neurological tissue [6]. This resulted in an attenuated phenotype characterized by a loss of viscerotropism, loss of neurotropism, and loss of mosquito competence. The French Neurotropic Vaccine (FNV) was derived from the WT French Viscerotropic Virus (FVV) by 128 serial passages in mouse brain [7]. The phenotype of FNV is similar to strain 17D, with a loss of viscerotropism and loss of mosquito competence, however, the strain displays enhanced neurotropism due to passage in mouse brain. While FNV was highly immunogenic, the vaccine was discontinued in 1981 due to the increased neuroinvasive properties of the virus, particularly in children. Genome sequencing showed that both LAVs differed from their respective parent WT virus by 20 amino acid substitutions (Table 1) that were spread throughout the structural and non-structural proteins, but the two WT-LAV pairs shared only two common amino acid substitutions at M-36 and NS4B-95 [8,9]. Studies of the RNA population diversity of YF LAVs found that both 17D and FNV are less genetically diverse compared to their parent viruses, although 17D is considerably lower in genetic diversity than FNV [8,9]. As both WT and vaccine strain viruses reach similar infectivity titers in cell culture and display similar multiplication kinetics in the cell types used for these studies, these findings were not believed to be due to differences in replication rates of the viruses. Similarly, both LAVs are relatively resistant to the antiviral Ribavirin compared to their WT parental strains [10]. The lack of genetic diversity and relative resistance to Ribavirin phenotypes are thought to contribute to the attenuation and genetic stability of the LAVs.

In the 1960s, it was shown that passaging WT Asibi virus in HeLa cells resulted in an attenuated phenotype after only five to six passages (Asibi HeLa p6 virus) [11,12]. Specifically, the resulting virus had the same phenotype as strain 17D, displaying a loss of viscerotropism, loss of neurotropism, and loss of mosquito competence [11,12,13,14]. A subsequent study showed that WT FVV was also attenuated for mouse neuroinvasiveness following five to six passages in HeLa cells [15]. The genome of Asibi HeLa p6 virus was sequenced by Sanger sequencing and compared to the parent Asibi strain. Asibi HeLa p6 had only 10 amino acid substitutions distinguishing it from the parental WT Asibi virus [15]. The majority of the substitutions occurred in the envelope (E) protein but there were also several substitutions in NS proteins. In the current study, we decided to determine the genomic sequence of HeLa passaged FVV to understand the molecular basis of attenuation of HeLa passaged FVV and compare to that of Asibi HeLa p6 virus. 

## 2. Materials and Methods

To prevent repeating all the studies undertaken previously and potential passaging artifacts, we used the same virus as studied by Dunster et al. in 1990 [15]. FVV is a wild-type YFV strain isolated from Francois Mayali in Senegal in 1927. FVV HeLa passage six was not available and so FVV HeLa p5 was used, which was shown to have an attenuated phenotype in mice similar to FVV HeLa p6 [15]. RNA was extracted from ampoules of FVV HeLa p5 and WT non-HeLa passaged FVV using the QIAamp viral RNA isolation kit and sequenced on an Illumina NextSeq 550 instrument by the UTMB Sequencing Core. The FVV virus used in the study was passaged minimally in Vero cells and the genome was used as a reference, along with previously published FVV and FNV genomes to align the reads using the Bowtie2 program, and PCR duplicates were removed using Picard Tools MarkDuplicates. FVV had an average coverage of 6658 reads while FVV HeLa p5 had an average coverage of 3951 reads. Samples were downsampled to the lowest mean coverage (3951) and Shannon Entropy was calculated in R Studio, as described previously, and statistics were calculated in GraphPad Prism [10]. 

## 3. Results

The consensus sequence for the FVV HeLa p5 (Genbank MZ285906) virus was aligned to the published WT FVV and FNV genome sequences as well as the newly sequenced FVV sample (Genbank U21056.) [9]. Seven nucleotide differences were found, encoding five amino acid substitutions at E-D155A, E-K331R, E-I412V, NS2A-T105A, and NS4B-V98I (Table 1). 

These coding changes were all comprised of nucleotide changes at either the first or second position in the codon, indicating selection pressure. For comparison, the 20 coding changes between strains FVV and FNV were also included in Table 1. Like strain FNV, the coding changes in FVV HeLa p5 were also spread across the viral genome, in both structural and non-structural genes. It was also of interest to see several conserved substitutions between the vaccine strain and FVV HeLa p5 at residues E-331, NS2A-105, and NS4B-98. Residue E-155 is also a conserved substitution, but between Asibi to Asibi HeLa p6, and is located in Domain I of the E protein. This residue is part of a YFV type specific epitope [16]. This domain is also involved in stability of the E protein as well as conformational changes of the E protein during virus particle maturation [17]. Interestingly, 17D-204 substrain vaccines do not have the glycosylation site while 17D-WHO substrain viruses do. However, comparison of immunity, including neutralizing antibodies, by different 17D vaccine substrains shows no differences [18]. Residue E-331 is a conserved substitution between FVV to FNV as well as Asibi to Asibi HeLa p6, but not Asibi to 17D, and is located in Domain III. This domain is involved in receptor recognition and contains important epitopes for viral neutralization and mutations in this region, which are known to impact viral virulence [17]. The structure of YFV domain III suggests that mutation of E-331 would not affect the structure of the domain and so not alter the immune response induced by the virus [19]. Residue E-412 is not a conserved substitution and is located in the “stem” region of the E protein [20]. Residue NS2A-105 is a conserved substitution between FVV to FNV. NS2A is a hydrophobic, non-membrane bound protein involved in virus assembly as well as the modulation of the immune response to the virus [21]. The final substitution, NS4B-98, is shared by FVV to FNV and Asibi to Asibi HeLa p6, but not Asibi to 17D. NS4B is an integral membrane protein that has many functions including playing a role in viral replication, where it may be involved in dsRNA disassociation as well as acting as an IFN-α/β antagonist [21,22,23]. The residue at NS4B-98 is in an area known to be involved in the interaction between NS4B and NS1 [24]. Thus, two residues (E-331 and NS4B-98) are conserved substitutions in the attenuation of two closely related WT strains of YFV (Asibi and FVV) by passage in HeLa cells, and NS2A-105 is a conserved substitution for attenuation of WT FVV by passage in HeLa cells and mouse brain (FNV). This conservation highlights residues, or regions of viral proteins, that could be very important in YF attenuation. Interestingly, the NS2A-T105A substitution is also one of the residues in a motif “L**T**L” to “M**A**F” that distinguishes WT West African genotype strains from East, East/central Africa, and Angola genotype strains, suggesting that this is an important residue in the phenotype of these strains [25]. However, at present, little is known about the phenotypic differences that distinguish the seven genotypes of WT YFV.

**Figure 1:**
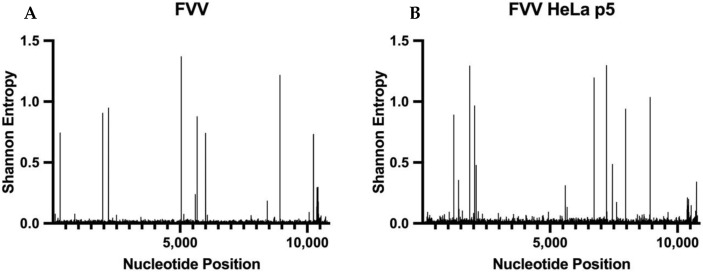
Shannon Entropy Values for FVV HeLa p5 (**A**) and FVV (**B**). FVV HeLa P5 displays statistically similar levels of Shannon Entropy compared to FVV.

Previous studies have shown that serial passage of WT Asibi to generate the attenuated strain 17D and WT FVV to generate the attenuated strain FNV was associated with decreased genetic diversity in terms of both Shannon entropy and Single Nucleotide Variants (SNVs) [8,9]. Shannon entropy is a calculation measuring the nucleotide variation at a specific location in the genome. A value for Shannon entropy is determined for every position in the YF genome with the higher values indicating more nucleotide variation at that position. The decrease in both genetic diversity and SNVs suggests a more stable virus with a higher fidelity replication complex, which would be beneficial for LAVs. As FVV HeLa p5 previously displayed an attenuated phenotype in mice, we hypothesized that Shannon entropy values would be expected to be lower than those of WT FVV. However, it was found upon analysis that WT FVV and FVV HeLa p5 were not significantly different in terms of Shannon entropy but FVV HeLa p5 did display several large peaks of Shannon entropy within the E gene (Figure 1). The SNVs greater than 1% of the RNA population were also similar between FVV and FVV HeLa p5 virus with nine and eight SNVs, respectively (Table 2), however, the SNVs were found in different positions across the genome. Five of the eight SNVs in FVV HeLa p5 virus clustered in the E gene with the remaining three in the NS genes (one each in NS2A, NS4B, and NS5), while six of the nine SNVs in FVV were in the NS genes and two in the 3′NCR. Seven of the eight SNVs identified in the FVV HeLa p5 population were coding changes, as shown in Table 2. Again, all SNV coding changes in FVV HeLa p5 were the result of nucleotide substitutions at either the first or second position in the codon, while only two of the three SNV coding changes for FVV were in those positions. The variants in the E protein of FVV HeLa p5 corresponded with the previously identified areas of high diversity with some of the SNVs occurring in large percentages of the population. Several of the SNVs were of note as they are WT variants (E-155, NS2A-105, and NS4B-98, all of which are conserved substitutions), suggesting that there is still the potential of reversion. 

**Figure 2:**
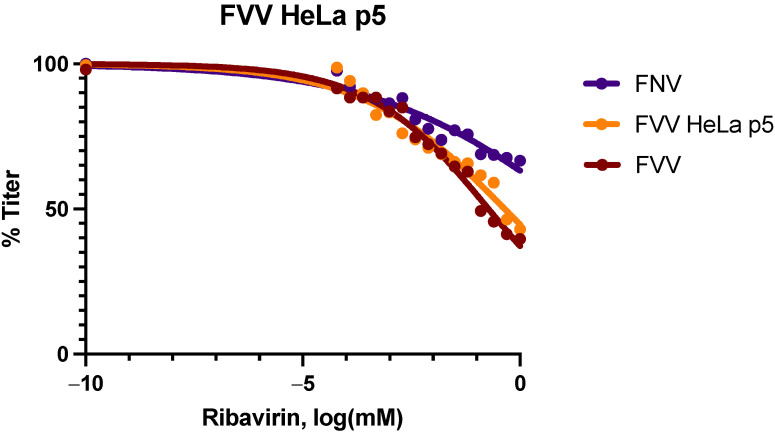
Ribavirin sensitivity curve for FVV HeLa p5 in Vero cells. FVV is in burgundy (0.20 µM, R^2^ = 0.96), FVV HeLa p5 (0.45 µM, R^2^ = 0.93) is in yellow, and FNV (17.31 µM, R^2^ = 0.79) is in purple.

Since previous studies had shown that 17D and FNV vaccine strains were significantly more resistant to the antiviral Ribavirin than their respective WT parental strains Asibi and FVV [10], correlating with the viral diversity of the viruses, a Ribavirin sensitivity assay was performed to confirm the findings that WT FVV and FVV HeLa p5 were not significantly different in terms of genetic diversity. FVV HeLa p5 virus was compared to FVV and FNV using the same assays as reported previously [10]. The 50% inhibitory concentration (IC_50_) value for FVV (0.20 µM, R^2^ = 0.96) and FNV (17.31 µM, R^2^ = 0.79) was similar to what has been previously reported [10]. The IC_50_ for FVV HeLa p5 (0.45 µM, R^2^ = 0.93) was not found to differ significantly from FVV (P = 0.8667), using the Freidman test with multiple comparisons. Thus, FVV HeLa p5 virus displayed similar levels of Ribavirin sensitivity as its parent virus WT FVV, while FNV was relatively resistant to Ribavirin (Figure 2), supporting the genetic diversity data.

## 4. Discussion

Overall, these findings indicate that the attenuation process of FVV through passage in HeLa cells results in a different genotype and phenotype compared to LAVs 17D and FNV. These findings suggest the mechanism of attenuation of a HeLa passaged virus is different to that of 17D or FNV obtained via passage in chicken tissue and mouse brain, respectively. As we have reported previously, the hallmark of 17D is the genetic stability of the virus [8]. Both 17D and FNV strains display a reduction in genetic diversity, reduction in SNVs, and increased antiviral resistance to Ribavirin compared to their wild-type parental viruses, which are important characteristics of a LAV that will not revert to virulence. We cannot exclude the possibility that these properties are not linked to the attenuated phenotype of 17D and FNV LAVs, but these qualities are vital to 17D as they directly relate to the safety and stability of the vaccine. As FVV HeLa p5 is not a vaccine strain, it offers a very different perspective on YF attenuation. The goal of this study was to explore the attenuation process behind HeLa passaged FVV virus with the objective of comparing it with that of 17D and FNV. The SNVs and Shannon entropy values of FVV Hela p5 virus suggest that, while the virus is attenuated for mouse neuroinvasion, it does not display the genetic stability characteristic of 17D and FNV LAVs. That reduced genetic diversity is not necessary for attenuation of virulence was a novel finding for YFV, but has been recently reported for another flavivirus. A study recently found that a similar Shannon entropy pattern was seen between WT Japanese Encephalitis virus (JEV) strain SA14 and its LAV strain SA-14-14-2 derived by passage in primary hamster kidney cells [26]. Thus, not surprisingly, there is more than one approach to attenuation of flaviviruses. 

To date, the study of attenuated strains of YFV with regards to viral diversity has been mostly limited to the LAV strains 17D and FNV. While the study of these LAVs allowed researchers to better understand the characteristics of safe and stable vaccine strains, much can also be learned from an attenuated strain of YFV derived by a different mechanism. The findings of this study indicate that FVV HeLa p5 is the first attenuated strain of YFV that displays levels of viral diversity similar to WT YFV while still maintaining an attenuated phenotype in mice [15]. Due to the novel nature of these findings, more studies are warranted to understand the mechanism of HeLa-passaged YFV attenuation in detail.

## Figures and Tables

**Table 1:** Coding Changes between FVV, FNV, and FVV HeLa p5.

Nucleotide	FVV	FVV HeLa p5	FNV	NT in Codon	Residue	FVV	FVV HeLa p5	FNV
239	T	T	C	2nd	C-80	V	V	A
268	A	A	G	1st	C-90	S	S	G
436	G	G	A	1st	prM-25	V	V	M
736	C	C	T	1st	M-36	L	L	F
882	C	T	C	3rd	E-9	R	R	R
1016	C	C	T	2nd	E-54	A	A	V
1319	A	C	A	2nd	E-155	D	**A**	D
1454	C	C	A	2nd	E-200	T	T	K
1600	A	A	G	1st	E-249	N	N	D
1847	A	G	G	2nd	E-331	K	**R**	R
2089	A	G	A	1st	E-412	I	**V**	I
3634	A	A	G	1st	NS2A-82	I	I	V
3703	A	G	G	1st	NS2A-105	T	**A**	A
4240	T	T	T	1st	NS2B-60	S	S	A
5027	T	T	A	2nd	NS3-192	M	M	K
6409	T	T	C	3rd	NS4A-29	F	F	L
6725	A	A	T	2nd	NS4A-135	Y	Y	F
7053	A	A	G	3rd	NS4B-95	I	I	M
7060	G	A	A	1st	NS4B-98	V	**I**	I
7262	C	C	T	2nd	NS4B-165	A	A	V
8291	T	T	C	2nd	NS5-258	I	I	T
8522	G	G	A	2nd	NS5-335	R	R	K
8799	C	T	C	3rd	NS5-427	V	V	V
9109	T	T	C	1st	NS5-531	F	F	L

**Table 2:** Single nucleotide variants (SNVs) for FVV and FVV Hela p5 virus. **A**: SNVs in FVV HeLa p5. Red denotes residue distinguishing FVV and FNV. Blue denotes residue distinguishing Asibi and Asibi HeLa p6. **B**: SNVs in FVV.

**A**							
CDS Position	HeLa P5	Variant	NT in Codon	Residue	HeLa P5	Variant	%
1117	C	A	1st	E-373	G	R	7.07
1319	**C**	**A**	**2nd**	**E-155**	**A**	**D**	1.34
1727	C	A	2nd	E-291	T	K	2.41
1823	A	G	2nd	E-323	K	R	17.66
1924	T	A	1st	E-357	S	T	5.78
3703	**G**	**A**	**1st**	**NS2A-105**	**A**	**T**	**1.68**
7060	**A**	**G**	**1st**	**NS4B-98**	**I**	**V**	1.01
9852	A	G	3rd	NS5-778	L	L	1.30
**B**							
CDS Position	FVV	Variant	NT in Codon	Residue	FVV	Variant	%
174	A	G	3rd	C-58	G	G	11.41
5490	T	G	3rd	NS3-346	S	R	2.50
5562	T	C	3rd	NS3-370	I	I	15.91
5895	T	C	3rd	NS3-481	P	P	12.54
8320	A	G	1st	NS5-268	T	A	1.84
10137	T	C	3rd	NS5-873	R	R	11.70
10142	G	A	2nd	NS5-875	R	Q	11.87
10276	G	C	N/A	3′UTR	N/A	N/A	1.83
10314	T	C	N/A	3′UTR	N/A	N/A	1.46

## Data Availability

The genome of FVV Hela p6 virus can be found in Genbank (MZ285906).

## References

[1] Monath T.P., Vasconcelos P.F. (2015). Yellow fever. J. Clin. Virol..

[2] Hamlet A., Jean K.K., Perea W., Yactayo S., Biey J., Van Kerkhove M., Ferguson N., Garske T. (2018). The seasonal influence of climate and environment on yellow fever transmission across Africa. PLoS Negl. Trop. Dis..

[3] Chippaux J.-P., Chippaux A. (2018). Yellow fever in Africa and the Americas: A historical and epidemiological perspective. J. Venom. Anim. Toxins Incl. Trop. Dis..

[4] Hamrick P.N., Aldighieri S., Machado G., Leonel D.G., Vilca L.M., Uriona S., Schneider M.C. (2017). Geographic patterns and environmental factors associated with human yellow fever presence in the Americas. PLoS Negl. Trop. Dis..

[5] Tsegaye M.M., Beyene B., Ayele W., Abebe A., Tareke I., Sall A., Yactayo S., Shibeshi M.E., Staples E., Belay D. (2018). Sero-prevalence of yellow fever and related Flavi viruses in Ethiopia: A public health perspective. BMC Public Health.

[6] Theiler M., Smith H.H. (1937). The use of yellow fever virus modified by in vitro cultivation for human immunization. J. Exp. Med..

[7] Lloyd W., Penna H.A. (1933). Studies on the Pathogenesis of Neurotropic Yellow Fever Virus in Macacus Rhesus 1. Am. J. Trop. Med. Hyg..

[8] Beck A., Tesh R.B., Wood T.G., Widen S.G., Ryman K.D., Barrett A.D.T. (2013). Comparison of the Live Attenuated Yellow Fever Vaccine 17D-204 Strain to Its Virulent Parental Strain Asibi by Deep Sequencing. J. Infect. Dis..

[9] Beck A.S., Wood T.G., Widen S.G., Thompson J.K., Barrett A.D.T. (2018). Analysis by deep sequencing of discontinued neurotropic yellow fever vaccine strains. Sci. Rep..

[10] Davis E., Beck A.S., Strother A.E., Thompson J.K., Widen S.G., Higgs S., Wood T.G., Barrett A.D.T. (2019). Attenuation of live-attenuated yellow fever 17d vaccine virus is localized to a high-fidelity replication complex. mBio.

[11] Hardy F.M. (1963). The growth of Asibi strain yellow fever virus in tissue cultures: I. Sensitivity and capacity of tissue cultures. J. Infect. Dis..

[12] Hearn H.J., Chappell W.A., Demchak P.E.T.E.R., Kominik J.W. (1966). Attenuation of aerosolized yellow fever virus after passage in cell culture. Bacteriol. Rev..

[13] Barrett A.D.T., Monath T.P., Cropp C.B., Adkins J.A., Ledger T.N., Gould E.A., Schlesinger J.J., Kinney R.M., Trent D.W. (1990). Attenuation of wild-type yellow fever virus by passage in HeLa cells. J. Gen. Virol..

[14] Miller B.R., Adkins D. (1988). Biological characterization of plaque-size variants of yellow fever virus in mosquitoes and mice. Acta Virol..

[15] Dunster L.M., Wang H., Ryman K.D., Miller B.R., Watowich S.J., Minor P.D., Barrett A.D. (1999). Molecular and Biological Changes Associated with HeLa Cell Attenuation of Wild-Type Yellow Fever Virus. Virology.

[16] Ryman K.D., Barrett A.D., Schlesinger J.J., Weir R.C., Ledger T.N. (1997). Yellow fever virus envelope protein has two discrete type-specific neutralizing epitopes. J. Gen. Virol..

[17] Zhang X., Jia R., Shen H., Wang M., Yin Z., Cheng A. (2017). Structures and functions of the envelope glycoprotein in flavivirus infections. Viruses.

[18] Juan-Giner A., Kimathi D., Grantz K.H., Hamaluba M., Kazooba P., Njuguna P., Fall G., Dia M., Bob N.S., Monath T.P. (2021). Immunogenicity and safety of fractional doses of yellow fever vaccines: A randomised, double-blind, non-inferiority trial. Lancet.

[19] Volk D.E., May F.J., Gandham S.H., Anderson A., Von Lindern J.J., Beasley D.W., Barrett A.D., Gorenstein D.G. (2009). Structure of yellow fever virus envelope protein domain III. Virology.

[20] Lu X., Xiao H., Li S., Pang X., Song J., Liu S., Cheng H., Li Y., Wang X., Huang C. (2019). Double lock of a human neutralizing and protective monoclonal antibody targeting the yellow fever virus envelope. Cell Rep..

[21] Chen S., Wu Z., Wang M., Cheng A. (2017). Innate immune evasion mediated by flaviviridae non-structural proteins. Viruses.

[22] Chambers T.J., McCourt D.W., Rice C.M. (1989). Yellow fever virus proteins NS2A, NS213, and NS4B: Identification and partial N-terminal amino acid sequence analysis. Virology.

[23] Green A.M., Beatty P.R., Hadjilaou A., Harris E. (2013). Innate immunity to dengue virus infection and subversion of antiviral responses. J. Mol. Biol..

[24] Zmurko J., Neyts J., Dallmeier K. (2015). Flaviviral NS4b, chameleon and jack-in-the-box roles in viral replication and pathogenesis, and a molecular target for antiviral intervention. Rev. Med. Virol..

[25] Von Lindern J.J., Aroner S., Barrett N.D., Wicker J.A., Davis C.T., Barrett A.D.T. (2006). Genome analysis and phylogenetic relationships between east, central and west African isolates of Yellow fever virus. J. Gen. Virol..

[26] Davis E.H., Beck A.S., Li L., White M.M., Greenberg M.B., Thompson J.K., Widen S.G., Barrett A.D.T., Bourne N. (2021). Japanese encephalitis virus live attenuated vaccine strains display altered immunogenicity, virulence and genetic diversity. NPJ Vaccines.

